# A Characteristic Electrocardiographic Manifestation Suggests Hypercalcemia

**DOI:** 10.3390/diagnostics15233034

**Published:** 2025-11-28

**Authors:** Suoling Zhai, Qingsong Zhao, Juan Wang, Li Li, Jiongyang Chen, Zhi Zhang

**Affiliations:** Shanghai General Hospital, Shanghai Jiao Tong University School of Medicine, Shanghai 200080, China; drzhaishgh@hotmail.com (S.Z.); es14790946811@163.com (Q.Z.); wjuan920@126.com (J.W.); annylish@126.com (L.L.)

**Keywords:** hypercalcemia, T wave, QTm shortening, QT shortening, QTc shortening

## Abstract

**Background**: This study aims to investigate the electrocardiographic (ECG) alterations associated with hypercalcemia, focusing on characteristic T-wave alterations as potential diagnostic indicators. **Methods**: Utilizing a retrospective observational design, we analyzed ECG data from 64 hypercalcemic patients and a control group of 956 normocalcemic individuals. **Results**: Our findings reveal that 78.13% of hypercalcemic patients exhibited characteristic T-wave alterations, compared to only 14.64% in the control group (*p* < 0.001). Additionally, hypercalcemic patients demonstrated significantly shorter mean QT, QTc, and QTm intervals (QT: 340.5 ± 15.4 vs. 380.6 ± 75.5, QTc: 404.2 ± 78.4 vs. 415.7 ± 57.1, QTm: 226.6 ± 23.4 vs. 270.6 ± 27.0; all *p* < 0.01). Notably, the sensitivity, specificity, and accuracy of T-wave alterations for diagnosing hypercalcemia were 78.12%, 97.28%, and 96.08%, respectively, outperforming QT shortening (sensitivity: 48.43%, specificity: 76.99%). **Conclusions**: These results indicate that characteristic T-wave alterations are not only prevalent but also provide a reliable diagnostic tool for hypercalcemia, suggesting that integrating ECG analysis into routine clinical practice may enhance early detection and management of this condition.

## 1. Introduction

Hypercalcemia is a clinical condition marked by elevated calcium levels in the bloodstream, which can lead to significant morbidity, including complications such as kidney stones, bone pain, and neurological disturbances [1,2]. This condition not only affects the quality of life of patients but also imposes substantial healthcare costs [3]. Current diagnostic modalities primarily rely on serum calcium measurements. Studies have already suggested that electrocardiogram (ECG) changes could serve as potential indicators of electrolyte imbalances, including those seen in hypercalcemia. In the previous literature, the effects of hypercalcemia on the ECG are primarily characterized by shortened QT intervals, elevated ST segments, and alterations in T waves [4]. Shortened QT intervals represent the most common ECG manifestation of hypercalcemia, resulting from accelerated myocardial cell repolarization due to elevated serum calcium levels. Hypercalcemia may also lead to ST segment elevation, a finding that must be carefully differentiated from acute myocardial infarction in clinical settings [5]. In patients with mild hypercalcemia, ECG changes may be subtle or limited to a slight shortening of the QT interval. In contrast, patients with moderate to severe hypercalcemia often exhibit more pronounced abnormalities, including marked QT interval shortening, ST segment elevation, and T-wave inversion [6]. The presence of these ECG changes underscores the necessity for prompt therapeutic intervention. Therefore, early recognition and management of hypercalcemia are critically important in clinical practice. Clinicians should be well-versed in the common etiologies of hypercalcemia and its associated ECG findings to enable timely diagnosis and appropriate treatment. However, those findings including QT shortening had poor predication for hypercalcemia or were not easy to be detected or calculated. Traditionally, the ECG diagnosis of hypercalcemia has traditionally relied on QT interval shortening as the key criterion. However, nearly in half of patients with elevated serum calcium levels, both the QT and corrected QT (QTc) intervals remained within normal ranges. Therefore, the ECG manifestations have not been fully explored, representing a significant gap in the literature [7,8].

Occasionally, Dr Zhai found that specific alterations in T-wave morphology (characterized by V1–V3 ST segment disappearing and leftward shift in the T-wave peak) maybe have been associated with the increase in calcium levels, which may reflect underlying cardiac dysfunction. Such findings suggest that ECG could become an easier way to diagnose hypercalcemia, potentially aiding in the identification of patients at risk of complications due to elevated calcium levels.

This research employs a retrospective observational study design to explore the relationship between ECG changes and hypercalcemia. 6020 patients were screened and then hypercalcemic patients and a control group of normocalcemic individuals were compared. The study aims to provide a comprehensive evaluation of ECG characteristics associated with elevated calcium levels. The focus is particularly on T-wave alterations, which have been previously identified as significant indicators of electrolyte imbalances [9]. The primary objective of this research is to assess the sensitivity and specificity of these ECG changes in diagnosing hypercalcemia, thereby enhancing the diagnostic accuracy and clinical management of affected patients.

## 2. Methods

Study Subjects: A retrospective review was performed of 6020 patients who attended the outpatient clinics but mainly from departments such as nephrology, cardiology, emergency department, or were admitted to wards of Shanghai General Hospital (formerly Shanghai First People’s Hospital) between January 2020 and January 2025. The inclusion criteria were as follows: availability of electrocardiogram (ECG) records and serum calcium measurements within 24 h before or after the ECG examination, and the patient’s myocardial enzyme monitoring results are normal, and the blood potassium/sodium levels are within the normal range. Ultimately, 64 patients with hypercalcemia were identified and included in the experimental group based on their 12-lead ECGs. During the same period, 956 patients with normal serum calcium levels were selected to the control group. Both groups were analyzed for characteristic T-wave alterations on ECG (notably, V1–V3 ST segment disappearing and leftward shift in the T-wave peak), as well as QT, QTc, and QTm interval shortening. (QT shortening: The time from the beginning of the Q wave to the end of the T wave has been shortened. QTc shortening: The quantitative value of QT/√RR has been decreased. QTm shortening: The distance from the starting point of the QRS wave to the peak of the T wave has been shortened).

Characteristic T-wave alterations in hypercalcemic patients were primarily defined as follows: (1) the ST segment is absent in most precordial leads, most prominently in leads V1–V3; occasionally, shortened ST segments may be observed in some left lateral precordial leads; (2) a leftward shift in the T-wave peak is evident across all 12 leads; and (3) the internal angle subtended by the ascending limb of the T wave is greater than that subtended by the descending limb. QTm ≤ 240 ms, QT ≤ 340 ms, and QTc ≤ 340 ms were considered as indicators of interval shortening. ECG measurements (QT, T-peak timing, ST amplitude) were performed independently by two cardiologists blinded to laboratory results. Disagreement > 10 ms for intervals or >0.05 mV for ST amplitude was adjudicated by a third reader.

Diagnostic criteria for hypercalcemia: normal serum calcium concentration ranges from 2.25 to 2.58 mmol/L. Mild hypercalcemia is defined as 2.5–3.0 mmol/L, moderate hypercalcemia as 3.0–3.5 mmol/L, severe hypercalcemia as greater than 3.5 mmol/L, and hypercalcemic crisis as exceeding 3.75 mmol/L.

### Statistical Analysis

Categorical variables were presented as frequency rates and percentages and were compared by chi-square or Fisher exact test. Continuous variables were presented as Means ± SD. Means for continuous variables were compared using independent group *t*-test or ANOVA when the data were normally distributed; otherwise, the Mann–Whitney test was used. Two-sided *p* < 0.05 was considered statistically significant. All statistical analyses were performed with SPSS software (version 24.0, IBM). Sensitivity was calculated to evaluate the ability of the EKG changes (characteristic T-wave alterations) to correctly identify hypercalcemic cases. The formula used for sensitivity calculation was as follows:

$$Sensitivity=\frac{True\;Positives\;(TP)}{True\;Positives\;(TP)+False\;Negatives\;(FN)}$$

Specificity was calculated to evaluate the ability of the EKG alterations to correctly identify true negative cases. The formula used for specificity calculation was as follows:

$$Specificity=\frac{True\;Negatives\;(TN)}{True\;Negatives\;(TN)+False\;Positives\;(FP)}$$

The formula used for accuracy calculation was as follows:

$$Accuracy=\frac{True\;Positives\;\left(TP\right)+True\;Negatives\;(TN)}{True\;Positives\;\left(TP\right)+False\;Negatives\;\left(FN\right)+True\;Negatives\;(TN)+False\;Positives\;(FP)}$$

- True Positives (TP) were defined as subjects correctly identified as positive by both the EKG alterations and the blood test.
- False Negatives (FN) were defined as subjects incorrectly identified as negative by the EKG alterations but positive by the blood test.
- True Negatives (TN) were defined as subjects correctly identified as negative by both the EKG alterations and the blood test.
- False Positives (FP) were defined as subjects incorrectly identified as positive by the EKG alterations but negative by the blood test.

## 3. Results

**Figure 1:**
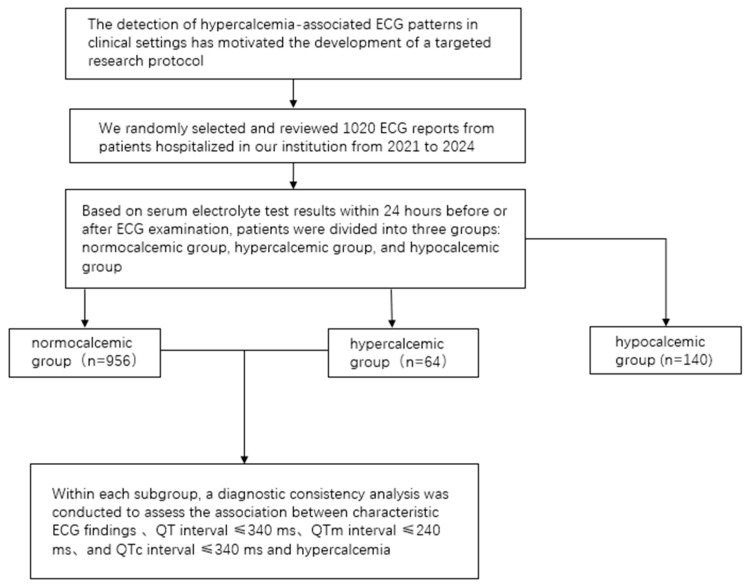
The schematic diagram of the study operation.

Among the 64 patients in the hypercalcemia group, there were 35 males and 29 females, with a mean age of 62 ± 17 years. In the control group of 956 patients, 458 were male and 498 were female, with a mean age of 58 ± 16 years. Among the 64 patients diagnosed with hypercalcemia, 54 had underlying conditions closely associated with elevated serum calcium levels. Hyperparathyroidism, renal failure, and malignant tumors collectively accounted for 76.6% of these cases (Table 1). Figure 1 shows the schematic diagram of the study operation.

**Figure 2:**
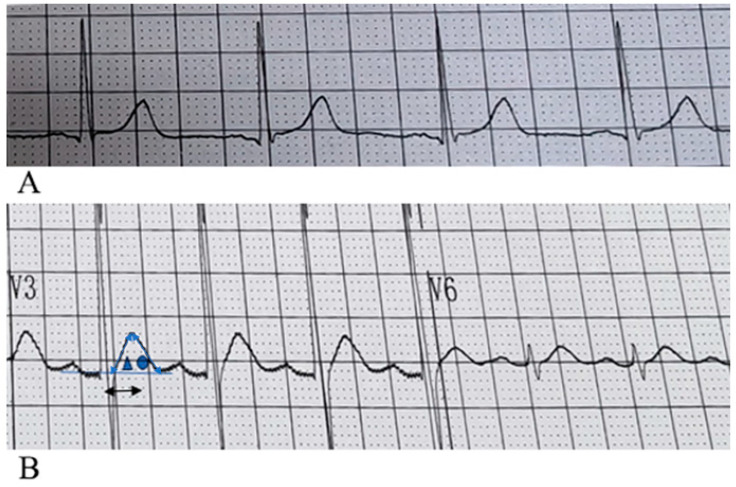
(**A**) Normal ST segment and T wave variations. The ST segment exists, and the ascending limb of the T wave is slow while the descending limb is steep. (**B**) Changes in ST segments and T waves in patients with hypercalcemia: In lead V3, ST disappears, and in lead V6, the ST segment shortens. The internal angle between the front branch of the T wave and the horizontal baseline ▲ is greater than that between the back branch and the horizontal baseline ●. The peak of the T wave appears earlier. The arrow ↔ in the figure represents QTm, which is 160 ms (the distance from the starting point of QRS to the vertex of the T wave). The shortening is ≤240 ms. The peak of the T wave moves leftward and approaches the QRS wave, which is a typical pattern change in hypercalcemia.

**Figure 3:**
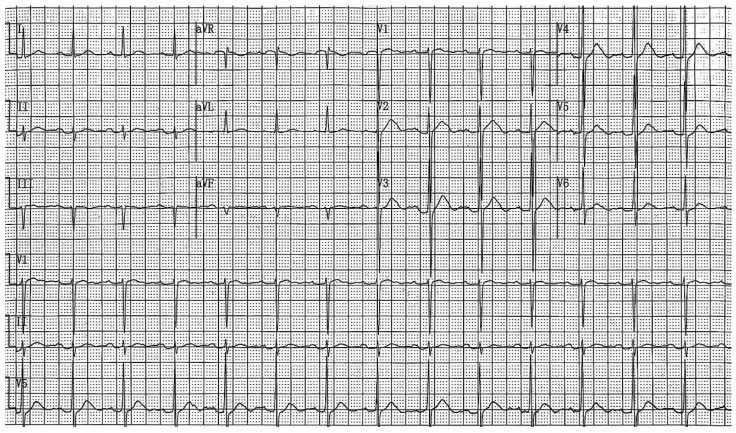
Hypercalcemic pattern 12-lead electrocardiogram: patient data included a heart rate of 86 beats per minute and a total blood calcium level of 3.44 mmol/L. The electrocardiogram showed a ventricular rate of 85 beats per minute, with a QT interval of 354 ms and a corrected QT interval (QTc) of 421 ms. In the 12-lead ECG, the T-wave peak was markedly shifted to the left in leads V1–V6, and the angle of the ascending limb of the T wave was steeper than that of the descending limb. The ST segment disappeared in leads V1–V3, and the mean QT interval (QTm) was 220 ms.

**Figure 4:**
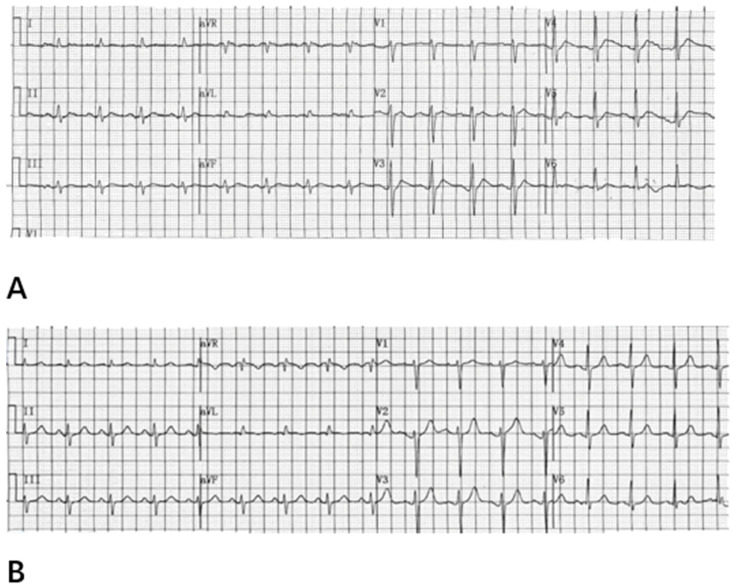
(**A**) Presents an electrocardiogram shows the 12-lead surface electrocardiogram (ECG) of the same patient with hypercalcemia. The blood calcium level was 3.49 mmol/L. The ECG reveals sinus tachycardia at a rate of 101 beats per minute, with a QT interval of 300 ms and a corrected QT (QTc) interval of 389 ms. The internal angle between the ascending limb of the T wave is larger than that of the descending limb, indicating a shortened maximum T-wave peak interval (QTm) of 160 ms and a leftward shift in the T wave peak. Additionally, the ST segments are absent in leads V1–V3, while they remain visible in other leads. (**B**) presents the 12-lead combined surface ECG after serum calcium levels returned to normal. The ECG demonstrates sinus rhythm at a rate of 98 beats per minute, with prolonged QT (342 ms), QTc (437 ms), and QTm (280 ms) intervals compared to the hypercalcemic state. The peak of the T wave shifts to the right. The ST segment of leads V1–V3 reappears.

By analyzing the 12-lead surface electrocardiogram (ECG) patterns of 64 patients with hypercalcemia, we identified characteristic T-wave alterations in most cases: (1) the ST segment is absent in most precordial leads, most prominently in leads V1–V3; occasionally, shortened ST segments may be observed in some left lateral precordial leads; (2) a leftward shift in the T-wave peak is evident across all 12 leads; and (3) the internal angle subtended by the ascending limb of the T wave is greater than that subtended by the descending limb. The electrocardiograms of patients with normal blood calcium levels and those with hypercalcemia, showing typical T-wave alterations, are presented in Figure 2 and Figure 3, respectively. The electrocardiogram changes associated with hypercalcemia and following calcium correction in the same patient are illustrated in Figure 4. We compared QT, QTc, and QTm between the hypercalcemic and normocalcemic groups. (Table 2). Moreover, we calculated the proportions of characteristic T-wave changes, QTm ≤ 240 ms, QT ≤ 340 ms, and QTc ≤ 340 ms in the hypercalcemia group and the normocalcemia group (Table 3).

**Table 2:** Comparison of QT, QTc, and QTm between hypercalcemic and normocalcemic groups.

	Hypercalcemia	Normocalcemia	*p* Value
QT (ms)	340.5 ± 15.4	380.6 ± 75.5	<0.01
QTc (ms)	404.2 ± 78.4	415.7 ± 57.1	<0.01
QTm (ms)	226.6 ± 23.4	270.6 ± 27.0	<0.01

**Table 3:** Proportions of characteristic T-Waves alteration, QTm ≤ 240 ms, QT ≤ 340 ms, and QTc ≤ 340 ms in hypercalcemic vs. normocalcemic groups.

	Hypercalcemia	Normocalcemia	*p* Value
Characteristic T-Waves alteration	78.13% (50/64)	2.7% (26/956)	<0.001
QT ≤ 340 ms	48.44% (31/64)	18.7% (179/956)	<0.001
QTc ≤ 340 ms	10.94% (7/64)	0.21% (2/956)	<0.001
QTm ≤ 240 ms	68.75% (44/64)	2.72% (26/956)	<0.001

## 4. Discussion

In this article, we identified and reported this specific electrocardiogram changes in patients with hypercalcemia, and compared these findings with those observed in patients with normal serum calcium levels. We summarized this change as (1) The ST segment is absent in most precordial leads, most prominently in leads V1–V3; occasionally, shortened ST segments may be observed in some left lateral precordial leads; (2) a leftward shift in the T-wave peak is evident across all 12 leads; and (3) the internal angle subtended by the ascending limb of the T wave is greater than that subtended by the descending limb, which may also be referred to as “Specific electrocardiogram for hypercalcemia” within the context of this paper. This study employs a retrospective observational design, analyzing a cohort of 64 hypercalcemic patients in comparison to a control group comprising 956 normocalcemic individuals, with the aim of assessing the sensitivity and specificity of ECG changes “leftward shift in the T-wave peak” in diagnosing hypercalcemia. We found it to have extremely high sensitivity and specificity in the diagnosis of hypercalcemia, and more importantly, it is easier to recognize than other characteristics previously reported, such as QT shortening.

In the past, electrocardiogram diagnosis of hypercalcemia was based solely on QT shortening, that is, QT ≤ 340 ms. Later, QTc ≤ 340 ms was set as the QT shortening standard [7,10,11]. However, we found that electrocardiographic specific T-wave changes also had a good effect in indicating hypercalcemia. Our observations regarding the disappearance of the ST segment and the leftward shift in the T-wave peak are consistent with previous reports. They have proposed that ST segments become shorter or disappear in patients with hypercalcemia, but they have not elaborated on this phenomenon [12,13]. According to our research, it is not difficult to find that although QTc shortening shows the highest specificity of 99.8% in diagnosing hypercalcemia and the specificity of QT shortening is 76.99%, the sensitivity of QT shortening is only 48.43%, and the sensitivity of QTc is even lower, at only 10.9%. Therefore, in the past, diagnosing hypercalcemia based on shortened QT and QTc would miss a considerable number of cases of hypercalcemia. Even with the QTm indicator, the sensitivity is only 64.06%, and the sensitivity based on the specific electrocardiogram morphology of hypercalcemia is 78.1% (95% CI: 65.8–87.4%), with a specificity of 97.28% (95% CI: 96.0–98.3%) (Table 4). In clinical practice, several factors can cause QT shortening, including fever, hyperkalemia, acidosis, acute myocardial injury, autonomic nervous system disorders, and ion channel diseases. As shown in Table 4, the isolated use of QT shortening as an indicator lacks sufficient sensitivity for the diagnosis of hypercalcemia. Therefore, there is a pressing need in clinical settings for an ECG indicator that is both specific and highly sensitive for detecting hypercalcemia. The hypercalcemia-specific ECG index we have summarized meets these criteria—it is easy for clinicians to learn, and demonstrates high sensitivity and specificity.

Hypercalcemia is a common clinical condition with diverse etiologies. According to recent studies, primary hyperparathyroidism (PHPT) is the leading cause of hypercalcemia among outpatients, accounting for the majority of cases [14]. In contrast, among hospitalized patients, hypercalcemia is most frequently associated with malignancies, comprising 54–65% of reported cases [2]. Hypercalcemia can not only cause cardiovascular abnormalities, but also lead to complications in multiple other organ systems. For example, hypercalcemia may result in neurological complications such as mental confusion, drowsiness, and even coma. Studies have demonstrated that the incidence of neurological complications is notably high among patients with severe hypercalcemia. In addition, hypercalcemia can cause gastrointestinal complications, including nausea, vomiting, constipation, and pancreatitis. Hypercalcemia-induced pancreatitis is relatively common in clinical practice and often presents with a severe clinical course [15,16,17].

Mechanism of ECG changes in hypercalcemia: relative hypertrophy of the left ventricle compared with the right ventricle causes the left ventricle to remain depolarized longer; consequently, when right ventricular depolarization is complete, the left ventricle may still be undergoing depolarization. Because right ventricular depolarization and repolarization occur earlier, ionic current changes are more prominent in the right precordial leads. Explanation for disappearance of the R component and related waveform changes: With elevated serum calcium, the extracellular membrane potential becomes more positive, increasing the transmembrane potential difference and lowering the threshold for potassium efflux. This promotes potassium efflux and initiates repolarization. Phase 2 of the ventricular action potential (the plateau) is primarily maintained by slow inward calcium current, which counterbalances inward chloride currents during phase 1; when intracellular/extracellular calcium concentrations rise, a sudden outward potassium current can disrupt the plateau and precipitate an early transition to phase 3, causing loss of the phase-2 plateau. On the surface ECG, phase 2 corresponds to the ST segment and phase 3 corresponds to the T wave. Because the loss of phase 2 is accompanied by an abrupt increase in outward potassium current, the resulting transient rise in repolarization voltage advances the timing of the T-wave peak, producing a characteristic leftward shift in the T-wave apex on the ECG so that the T-wave peak appears earlier and closer to the R wave [18].

Hypercalcemia is a common electrolyte disorder that can result in various abnormal electrocardiogram (ECG) findings. Its underlying mechanism is closely associated with the role of calcium ions in myocardial cells. Firstly, hypercalcemia shortens the action potential duration of myocardial cells, primarily by accelerating calcium ion influx, which leads to a reduction in the QT interval. These changes are typically reflected on the ECG as a shortened QT interval and alterations in the T-wave morphology. Secondly, hypercalcemia may also influence the repolarization phase of the cardiac cycle. Elevated calcium levels can interfere with potassium ion flux, resulting in early repolarization and J-point elevation [12]. These effects are commonly observed as J-point elevation and features consistent with early repolarization syndrome on the ECG. In addition, hypercalcemia may increase the excitability of myocardial cells and enhance the activity of ectopic pacemakers, thereby predisposing to arrhythmias such as tachycardia and atrial fibrillation, which can be reflected in abnormal electrocardiogram findings [19,20].

In many previously published articles on electrocardiogram (ECG) changes associated with hypercalcemia, although these changes have been described in various forms—such as patterns resembling myocardial infarction or Brugada syndrome—it is still evident that special change can occur. However, this specific change was not formally recognized or described by previous researchers. For example, some reports have suggested that hypercalcemia may manifest with ST-segment elevation, mimicking either myocardial infarction, early repolarization or Brugada-Type, our findings indicate that the disappearance of the ST segment and a leftward shift in T waves may better characterize the potential electrocardiographic features associated with hypercalcemia [18,21,22]. In the previous report, we found that the electrocardiogram of patients with high calcium levels showed the specific electrocardiographic manifestations we proposed for patients with hypercalcemia [12].

We found that the characteristic manifestations of hypercalcemia on the electrocardiogram was (1) The ST segment is absent in most precordial leads, most prominently in leads V1–V3; occasionally, shortened ST segments may be observed in some left lateral precordial leads; (2) a leftward shift in the T-wave peak is evident across all 12 leads; and (3) the internal angle subtended by the ascending limb of the T wave is greater than that subtended by the descending limb (see Figure 2). Among the 64 cases, significant leftward shifts in the T-wave peak were observed in leads V1–V3 in 50 cases, with the angle of the ascending limb of the T-wave being greater than or equal to that of the descending limb. 22 cases exhibited a leftward shift in the T-wave peak across all 12 leads. In some cases, widened QRS complexes as well as flattened or biphasic T-waves were observed; however, these findings were considered less specific. Some patients may present with concurrent electrolyte disturbances, such as hypokalemia.

On the electrocardiogram, shortening of the QT interval is primarily observed in cases with significantly elevated blood calcium levels. Common causes of QT interval shortening in clinical practice include genetic factors, electrolyte imbalances, and drug effects, among others, involving multiple pathophysiological mechanisms [11,23]. Genetic factors also represent a primary cause of QT interval shortening. This mechanism primarily operates through enhanced potassium ion efflux, which reduces the duration of the myocardial action potential [24,25,26,27]. However, in mild to moderate hypercalcemia, the QT interval typically does not shorten, which may be attributed to the fact that the phase 3 component represents a calcium-dependent transient increase in potassium ion conductance. During repolarization, potassium efflux continues for a certain duration and follows its intrinsic pattern. This phenomenon does not result in a shortening of the ventricular refractory period, a point that requires careful differentiation in clinical practice. When hypercalcemia coexists, potassium ions influence the amplitude of the T wave. The increase in potassium current during phase 3 is both dependent on calcium and transient in nature; therefore, the T-wave amplitude is often not markedly elevated. In an observation of 64 cases of hypercalcemia, none exhibited T-wave amplitude exceeding half the height of the R wave.

However, we acknowledge several limitations in our study that warrant discussion. The retrospective design and single-center approach may introduce selection bias, limiting the generalizability of our findings to broader populations. Additionally, the lack of longitudinal follow-up restricts our ability to assess the long-term outcomes of patients with hypercalcemia based on ECG findings. Future research should aim to include a larger, more diverse sample across multiple centers and employ prospective designs that allow for comprehensive assessments of the relationship between ECG changes and clinical outcomes over time. The relatively small number of hypercalcemia cases compared to controls (64 vs. 956) limits the power of subgroup analyses and raises the possibility of bias due to unmeasured center-specific factors. To address this limitation, we plan to conduct prospective, multicenter validation studies in subsequent research. These considerations have been incorporated into the limitations, and we are actively pursuing these methodological improvements in future work. Diagnosing hypercalcemia through specific electrocardiogram examination, excluding paced ECGs, adjusting for serum potassium and magnesium, and reviewing medication lists are needed. We emphasize that the ECG pattern increases suspicion for hypercalcemia but is not diagnostic without laboratory confirmation. Overall, while our study lays a strong foundation for future investigations, addressing these limitations will be crucial for validating the clinical utility of ECG in managing hypercalcemia effectively.

In summary, the specific electrocardiogram (ECG) features of hypercalcemia—such as a leftward shift in the peak of the T wave, the disappearance of the ST segment in leads V1–V3, along with a larger angle between the anterior branch of the T wave and the horizontal baseline compared to that of the posterior branch across all 12 leads, and a shortened QTm—can serve as specific indicators for the ECG diagnosis of hypercalcemia for clinical medical staff, with a specificity of 78.1%. We clarify that routine serum calcium testing is the diagnostic standard and is widely available, but ECG-based detection may be useful in scenarios where point-of-care testing is delayed or unavailable, such as prehospital settings, resource-limited environments, or for rapid triage in EDs when lab turnaround is prolonged. When combined with laboratory tests, these ECG changes can facilitate the timely detection of elevated blood calcium levels in patients.

## Figures and Tables

**Table 1:** Baseline characteristics of patients.

Baseline Characteristics	Hypercalcemia (*n* = 64)	Normocalcemia (*n* = 956)
Sex		
Male	35	458
Female	29	498
Age		
≤65	37	640
>65	27	316
Comorbidity		
Hyperparathyroidism	17	4
History of Malignant Tumor	16	11
Renal Failure	16	5
Acidosis	12	2
Bone Metabolism Disorder	6	28
None	10	923

**Table 4:** Accuracy, sensitivity and specificity between specific electrocardiographic patterns and three other ECG manifestations of hypercalcemia.

	Accuracy	Sensitivity	Specificity
Characteristic T-waves alteration	96.08%	78.12%	97.28%
95%CI	94.7–97.2%	65.8–87.4%	96.0–98.3%
QT ≤ 340 ms	75.2%	48.43%	76.99%
95%CI	72.4–77.8%	35.7–61.4%	74.1–79.7%
QTm ≤ 240 ms	92.65%	64.06%	94.56%
95%CI	90.9–94.2%	51.1–75.7%	92.9–96.0%
QTc ≤ 340 ms	94.22%	10.94%	99.8%
95%CI	92.6–95.6%	4.5–21.5%	99.1–99.97%

## Data Availability

The original contributions presented in this study are included in the article. Further inquiries can be directed to the corresponding authors.
